# Disease activity, quality of life and indirect costs of psoriatic arthritis in Poland

**DOI:** 10.1007/s00296-016-3514-3

**Published:** 2016-06-23

**Authors:** Paweł Kawalec, Krzysztof Piotr Malinowski, Andrzej Pilc

**Affiliations:** 1 Faculty of Health Science, Institute of Public Health, Jagiellonian University Medical College, Grzegórzecka 20, 31-531 Kraków, Poland; 2 Department of Neurobiology, Institute of Pharmacology, Polish Academy of Sciences, Kraków, Poland

**Keywords:** Quality of life, Indirect costs, Productivity loss, Psoriatic arthritis

## Abstract

The aim of the study was to assess the indirect costs, health-related quality of life and clinical characteristics of patients with psoriatic arthritis (PsA), measured using a PsA disease activity index in Poland. Additionally, we aimed to investigate the association between the activity, utility of PsA-affected patients and productivity loss in a Polish setting. A questionnaire survey was conducted to assess disease activity, as well as productivity loss, and a paper version of the EuroQoly-5D-3L questionnaire was used to assess productivity loss and the quality of life. Indirect costs were assessed with the human capital approach employing the gross domestic product (GDP) per capita, gross value added (GVA) and gross income (GI) per worker in 2014 in Poland and were expressed in Polish zlotys (PLN) as well as in euros. The correlation was presented using the Spearman correlation coefficient. Our analysis was performed on the basis of 50 full questionnaires collected. We observed a mean utility value of 0.6567. The mean number of days off work was 2.88 days per month, and mean on-the-job productivity loss was 24.1 %. Average monthly indirect costs per patient were €206.7 (864.01 PLN) calculated using the GDP; €484.56 (2025.46 PLN) calculated using the GVA; and €209.70 (876.56 PLN) calculated using the GI. PsA reduces the patients’ quality of life as well as their productivity loss associated with both absenteeism and presenteeism. Total indirect costs were negatively correlated with utility. The greater the disease activity, the lower the utility and the greater the indirect costs.

## Introduction

Psoriatic arthritis (PsA) is one of the most prevalent inflammatory joint diseases, occurring in 11.2 % (5.9–23.9 %) of patients with psoriasis (affecting approximately 2 % of the population) [1, 2]. The disease occurs when the immune system of a patient affected with psoriasis attacks its own joints, which leads to severe pain, severe joint destruction, mutilating arthritis and co-morbidities [3, 4].

Rheumatic diseases are a major cause of disability and generate significant socio-economic costs. People affected with PsA require lifelong treatment, which causes great direct costs to the national health insurance system. Severe pain and joint destruction reduce the quality of life, which is a huge burden for the patient; moreover, the disease can reduce the ability to work, can cause interruptions in employment and can decrease productivity. The value of lost productivity is an indirect cost, that is, the cost not due to expenses but due to the lack of work caused by disease.

The indirect costs that follow PsA are associated with two types of indirect costs (generated productivity loss): absence from work (absenteeism) and a decrease in the efficiency of work performed by a sick patient (presenteeism). Absenteeism refers to the number of days on sick leave, periods of unemployment caused by disease and leaving the labour market prematurely due to sickness (e.g. early disability pension or premature death). Presenteeism refers to a situation when a sick person is present at work, but his or her productivity is lower than average due to disease. Productivity loss due to lower efficiency of work has been studied since the early 1990s, but it is commonly excluded in studies on indirect costs owing to difficulties in the estimation of lower productivity.

Both absenteeism and presenteeism data can be obtained in a questionnaire survey using well-validated tools, such as the Work Productivity and Activity Impairment (WPAI) questionnaire. It is a standard analytical tool commonly used to measure impairments in work and activities [5]. Each category of indirect costs (absenteeism and presenteeism) can be calculated using two methods: the human capital approach (HCA) and the friction cost approach (FCA). The HCA converts the gross income (GI) that will not be generated in the future due to disease into real costs from a social perspective; hence, it assumes that a decrease in productivity is permanent (irreplaceable). The FCA takes into account the productivity loss until a new person is employed as a substitute for the sick one.

The EuroQol 5 dimensions 3 level version (EQ-5D-3L) questionnaire is a standardised instrument for use as a measure of the quality of life. It takes into account 5 most important aspects of life: mobility, self-care, housekeeping activities, pain and anxiety. For each domain, the patient can report the lack of problems (represented by digit 1), the occurrence of some problems (represented by digit 2) and the occurrence of many problems (represented by digit 3). A quality-of-life state is created by combining answers from all 5 domains, for example, the “1, 3, 2, 2, 2” state represents the lack of problems with mobility, many problems with self-care and some problems concerning the remaining 3 domains. Data obtained using the EQ-5D-3L questionnaire can be presented as the health state combined from all domains or can be converted into utility using population tariffs [6, 7].

## Objective

The aim of this study was to assess the indirect costs, health-related quality of life and clinical characteristics of patients with PsA, measured using a PsA disease activity index in Poland. The second aim was to investigate the association between disease activity and the quality of life and productivity loss of patients with PsA in a Polish setting.

## Materials and methods

A questionnaire-based, multicentre, cross-sectional survey was used to collect data on disease activity, health-related quality of life and productivity loss of patients with PsA in Poland. The survey was conducted from November until December 2015 (cut-off date, 31 December 2015). Volunteers aged 18 years or older with a diagnosis of PsA from randomly selected inpatient and outpatient medical centres in Poland were enrolled in the study. The randomisation was performed to get a sample of patients with the greatest representativeness for Polish population; the whole country was divided into 5 regions, and in each of these regions, the random selection of medical centres engaged in PsA therapy was performed; then, data obtained by doctors from these selected centres were collected.

The questionnaire consisted of two parts: the first one was designed to be completed by clinicians and the second one—by patients. Both parts were designated with a matching blank patient’s acronym/number assigned by clinicians. After enrolment to the study, clinicians completed data regarding the disease activity index as well as the current pharmacological regimen for each individual patient. In the second part of the questionnaire, respondents provided general demographic data (basic patient’s characteristics: age, sex, size of the place of residence, age at disease diagnosis, co-morbidities) as well as responded to a number of questions referring to the current health state related to PsA and reflecting self-assessment of disease activity; utility was assessed using the EQ-5D-3L questionnaire, including the visual analogue scale (VAS) assessment.

There are no specific scales validated to assess the disease activity of PsA. Therefore, disease activity was assessed with our own questionnaire developed on the basis of the Bath Ankylosing Spondylitis Disease Activity Index (BASDAI), which is used to measure the activity of ankylosing spondylitis [8]. In our assessment of disease activity, we considered the major symptoms of PsA such as tiredness, joint pain, discomfort in the areas tender to touch or pressure, as well as morning joint stiffness and its duration, which were assessed in the week preceding the day of completing the questionnaire. For each domain, patients reported the extent of a health problem on a scale from 0 to 10: the higher the value, the greater the activity of the disease. Additionally, for each individual patient, we assessed the overall disease activity, tiredness and effort, and joint pain due to the disease on the same scale, from 0 to 10. Some questions regarding direct medical costs associated with PsA therapy were also included such as a monthly range of out-of-pocket expenses on medical and nonmedical resources (e.g. private medical consultations, formal and informal care, and over-the-counter medication). A number of questions referring to productivity loss (presenteeism, absenteeism), impairment of usual activities, informal care and benefits taken from a social care institution according to the WPAI questionnaire were also implemented.

The HCA was used to estimate indirect costs due to absenteeism and presenteeism. Three macroeconomic indicators for Poland were considered: gross domestic product (GDP) per capita (equal to €10,688 or 44,677 PLN), gross value added (GVA) per worker (equal to €25,055 or 104,728 PLN) and GI per worker (equal to €10,843 or 45,325 PLN), presented in 2014 prices in euro (the exchange rate was 1 euro = 4.18 PLN) with a correction factor of 0.65 (the conventional mean value of output elasticity of labour according to the Cobb–Douglas function of production; this approach is suggested by the European Commission because increasing the amount of a single factor of production, with all the other factors of production constant, decreases the marginal output of production) [9, 10]. The GDP per capita is a commonly used measure of a country’s economic development; it is a standard method of calculations used on a regular basis as revealed in a literature. The GVA is an increase of value of production due to work performed by a human capital. It reflects true economic development in areas of production and is expressed as value per worker; hence, it does not consider economically inactive people. Finally, the GI is a macroeconomic indicator that best reflects the patient’s perspective regarding lost productivity; however, it does not consider several types of job contracts. Assessing indirect costs using the GI is particularly important for patients because it reflects lost earnings.

Continuous variables were summarised using means and standard deviations and medians with the first and the third quartiles due to skewness, while nominal variables were summarised using counts and percentages. The Spearman’s correlation coefficient was used to present the association between disease activity, quality of life, as well as absenteeism and presenteeism. Variables of at least ordinal scale were compared using the Mann–Whitney *U* test. *P* values of <0.05 indicated statistical significance. Statistical analyses were performed using JMP^®^ 9.0.0 and R^®^ 3.2.2 software.

## Results

We obtained 50 completed questionnaires from patients aged from 18 to 84 years (average age, 45.54 years; SD 13.89). The basic characteristics of the population are presented in Table 1.

**Table 1:** Basic characteristics of patients included in the study

Characteristic	Value for sample
Age (years)	45.5 (35.75–53.5)
Male	21 (42 %)
Age at onset (years)	36.5 (29–44)
Place of living	
City	43 (86 %)
Village	7 (14 %)

Clinical experts evaluated the activity of PsA for 49 of 50 patients; 24 patients (49 %) were shown to have disease remission (or very low activity) and 25 patients (51 %)—active disease. All patients provided data on disease activity on the VAS based on the modified BASDAI scale. Patients reported median values of: 40 % (Q1–Q3: 20–60 %) on the scale regarding tiredness; 40 % (Q1–Q3: 20–70 %) on the scale regarding pain with affected joints; 40 % (Q1–Q3: 10–60 %) on the scale regarding discomfort in the areas tender to touch or pressure; and 35 % (Q1–Q3: 10–60 %) on the scale regarding discomfort at the moment of waking up (joint stiffness) and during 30 min (15–60 min) of morning stiffness. Based on those values, we calculated a disease activity score of 3.1 (Q1–Q3: 1.48–4.68) (Table 2).

**Table 2:** Patient-reported and physician-reported disease activity

Domain	Disease activity assessed by expert clinician		*P* value
	Remission/mild	Active	
Tiredness	2 (1–3.75)	6 (4–7)	<0.0001
Pain	2 (1–2)	7 (5–8)	<0.0001
Discomfort from areas tender to touch or pressure	1.5 (1–4)	6 (4–7)	<0.0001
Morning joint stiffness	1.5 (0.25–2)	6 (4–7)	<0.0001
Morning joint stiffness duration (min)	15 (7.75–27.5)	60 (33.5–77.5)	<0.0001
Disease activity score	1.5 (0.9–2.4)	4.6 (3.4–5.4)	<0.0001

Median with the first and the third quartile

Data on the quality of life were also reported by all respondents, except for the quality of life measured on the VAS, which was missing for 1 patient.

Based on data from Table 3, the health states were created. The most common state was “1, 1, 1, 1, 1”, reported by 12 patients (24 %), followed by “2, 2, 2, 2, 2”, reported by 8 patients (16 %). The highest (worse) states observed were “3, 3, 3, 2, 2” and “3, 2, 3, 3, 2”, which occurred in 1 patient each (4 %).

**Table 3:** Number of patients reporting problems in specific life domains included in the EQ-5D-3L questionnaire

Life domain	Problems reported with specific life domains		
	No	Some	A lot
Mobility	27 (54 %)	20 (40 %)	3 (6 %)
Self-care	33 (66 %)	15 (30 %)	2 (4 %)
Usual activities	18 (36 %)	28 (56 %)	4 (8 %)
Pain	13 (26 %)	34 (68 %)	3 (6 %)
Anxiety	26 (52 %)	21 (42 %)	3 (6 %)

The mean utility was 0.6567 (SD 0.2738), the lowest calculated value was 0.004, and the highest calculated value was 1.

Patients reported between 0 and 3 consultations with a clinician in a month before completing the questionnaire (mean 1.22; SD 0.65), of which from 0 to 3 were private consultations (mean 0.51; SD 0.73). The mean cost of consultation was €17.42 (SD €16.51) or 72.8 PLN (SD 69.01). Of all working patients, 58 % spent from €0 to €23.92 (100 PLN) monthly on drugs prescribed by a clinician and none of them spent more than €143.54 (600 PLN) monthly. A similar amount of money was spent on dietary supplements and other medicines that were not prescribed by a clinician. The smallest amount of money was spent monthly on information materials about the disease: more than 90 % of the patients spent from €0 to €23.92 (100 PLN) and none of the patients spent more than €47.85 (200 PLN).

Data on employment status were collected for all patients, of which 32 (64 %) were currently working. Among all working patients, 27 (84 %) were on a full-time contract. Only 2 patients (4 %) were still studying and only 6 patients were on pension, of which 3 due to the disease. Five patients were retired and thus not considered as economically active. The average monthly number of days off work was 3.88 (SD 6.19), with the minimum and maximum values of 0 and 24, respectively. Most respondents (37.5 %) reported lack of days off work due to the disease. More than a half of the patients reported less than 2 days on sick leave in a month. The mean on-the-job productivity loss was 24.1 % (SD 22.27 %), which represents the extent of presenteeism. The minimal reported value was 0 % and the maximum—70 %. Data were presented for all working patients with PsA.

As a main analysis, we used the GDP per capita and obtained the monthly indirect costs of €67.4 (SD €116.75) or 281.73 PLN (SD 488.02 PLN) for absenteeism and of €139.31 (SD €128.92) or 582.32 PLN (SD 538.89 PLN) for presenteeism. Taking into account the GVA per worker, we obtained the costs of €158 (SD €273.69) or 660.44 PLN (SD 1144.02 PLN) for absenteeism and of €326.56 (SD €302.21) or 1365.02 PLN (SD 1263.24 PLN) for presenteeism. Considering the GI per worker, we obtained monthly indirect costs of €68.38 (SD €118.44) or 285.81 PLN (SD 495.08 PLN) for absenteeism and of €141.33 (SD €130.79) or 590.74 PLN (SD 546.69 PLN) for presenteeism. The average monthly indirect cost of both absenteeism and presenteeism per patient was €206.7 (SD €203.24) or 864.01 PLN (SD 849.54 PLN) calculated using GDP; €484.56 (SD €476.44) or 2025.46 PLN (SD 1991.52 PLN) calculated using the GVA; and €209.70 (SD €206.19) or 876.56 PLN (SD 861.87 PLN) calculated using the GI. Data were based on answers of 32 working patients with PsA. The cost of presenteeism constituted 67.4 % of the total indirect costs.

As a sensitivity analysis, the results without the correction factor (marginal productivity of labour) were also presented. The total annual indirect costs per patient were €318.01 (SD €312.68) or 1329.28 PLN (SD 1307 PLN) calculated using the GDP; €745.48 (SD €732.99) or 3116.11 PLN (SD 3063.9 PLN), calculated using the GVA; and €322.62 (SD €317.21) or 1348.55 PLN (SD 1325.95 PLN) calculated using the GI (Table 4).

**Table 4:** Monthly indirect costs (in euro) due to PsA

Cost	Correction factor	Macroeconomic indicator	Mean (SD)	Median (Q1–Q3)	Skewness
Absenteeism	Yes	GDP	67.4 (116.75)	18.98 (0–18.98)	2.35
		GVA	158 (273.68)	44.5 (0–44.5)	
		GI	68.38 (118.44)	19.26 (0–19.26)	
	No	GDP	103.69 (179.61)	29.2 (0–29.2)	
		GVA	243.07 (421.05)	68.46 (0–68.46)	
		GI	105.19 (182.22)	29.63 (0–29.63)	
Presenteeism	Yes	GDP	139.31 (128.92)	115.79 (57.89–115.79)	0.87
		GVA	326.56 (302.21)	271.43 (135.71–271.43)	
		GI	141.33 (130.79)	117.47 (58.73–117.47)	
	No	GDP	214.32 (198.33)	178.13 (89.07–178.13)	
		GVA	502.4 (464.94)	417.58 (208.79–417.58)	
		GI	217.42 (201.21)	180.72 (90.36–180.72)	
Total cost	Yes	GDP	206.7 (203.24)	134.29 (62.64–134.29)	1.39
		GVA	484.56 (476.44)	314.81 (146.84–314.81)	
		GI	209.7 (206.19)	136.24 (63.55–136.24)	
	No	GDP	318.01 (312.68)	206.61 (96.37–206.61)	
		GVA	745.48 (732.99)	484.33 (225.91–484.33)	
		GI	322.62 (317.21)	209.6 (97.76–209.6)	

The total indirect costs as well as their components (absenteeism and presenteeism) differed significantly between patients with disease in remission (or mild activity) and those with active disease, as assessed by an expert clinician (Table 5).

**Table 5:** Monthly indirect costs (in euro) due to PsA depending on disease activity assessed by an expert clinician

Cost	Correction factor	Macroeconomic indicator	Disease activity				*P* value
			Remission/mild		Active		
			Mean (SD)	Median (Q1–Q3)	Mean (SD)	Median (Q1–Q3)	
Absenteeism	Yes	GDP	18.98 (31.05)	0 (0–23.73)	149.24 (165.9)	70 (34.40–313.19)	0.0004*
		GVA	44.5 (72.78)	0 (0–55.62)	349.85 (388.91)	164.08 (80.65–734.19)	
		GI	19.26 (31.5)	0 (0–24.07)	151.41 (168.31)	71 (34.90–317.74)	
	No	GDP	29.2 (47.76)	0 (0–36.50)	229.6 (255.23)	107.68 (52.93–481.84)	
		GVA	68.46 (111.96)	0 (0–85.57)	538.24 (598.32)	252.43 (124.08–1129.53)	
		GI	29.63 (48.45)	0 (0–37.03)	232.93 (258.93)	109.24 (53.7–488.82)	
Presenteeism	Yes	GDP	88.22 (85.12)	57.89 (0–115.79)	254.73 (139.69)	260.52 (101.31–405.25)	0.0026*
		GVA	206.8 (199.55)	135.71 (0–271.43)	597.14 (327.47)	610.72 (237.5–950)	
		GI	89.5 (86.36)	58.73 (0–117.47)	258.43 (141.72)	264.3 (102.78–411.13)	
	No	GDP	135.72 (130.96)	89.07 (0–178.13)	391.89 (214.91)	400.8 (155.87–623.47)	
		GVA	318.16 (307)	208.79 (0–417.58)	918.68 (503.8)	939.57 (365.39–1461.54)	
		GI	137.69 (132.86)	90.36 (0–180.72)	397.58 (218.03)	406.61 (158.13–632.51)	
Total cost	Yes	GDP	107.2 (96.2)	76.87 (0–191.71)	403.972 (232.07)	402.41 (258.86–523.77)	0.0004*
		GVA	251.3 (225.5)	180.21 (0–449.42)	946.999 (544.02)	943.33 (606.82–1227.83)	
		GI	108.75 (97.59)	77.99 (0–194.49)	409.831 (235.44)	408.24 (262.61–531.36)	
	No	GDP	164.92 (147.99)	118.27 (0–294.94)	621.496 (357.03)	619.09 (398.24–805.8)	
		GVA	386.62 (346.93)	277.25 (0–691.41)	1456.92 (836.96)	1451.27 (933.57–1888.97)	
		GI	167.32 (150.14)	119.98 (0–299.22)	630.509 (362.21)	628.06 (404.02–817.48)	

The patients’ quality of life expressed as utility was correlated with absenteeism and presenteeism (a correlation of −0.537 and −0.682, with *P* values of 0.002 and <0.001, respectively, which indicates statistical significance). Utility showed a significant negative moderate-to-strong correlation with absenteeism, presenteeism and total indirect costs (correlation of −0.772; *P* value of <0.001). This suggests that a higher quality of life was associated with lower indirect costs. The disease activity score was significantly correlated with indirect costs (positive correlation of 0.618 for absenteeism, of 0.838 for presenteeism and of 0.864 for total costs; *P* values of <0.0001) and quality of life (negative correlation of −0.878; *P* value of <0.0001). The quality of life and disease activity were significantly correlated with presenteeism and absenteeism (Table 6).

**Table 6:** Correlations between the quality of life, disease activity and indirect costs

Disease activity	Outcome	Correlation	*P* value
Tiredness	Cost due to absenteeism	0.6221	0.0001*
Pain		0.5352	0.0016*
Tenderness to touch or pressure		0.5134	0.0027*
Morning joint stiffness		0.4604	0.0080*
Morning joint stiffness duration		0.4815	0.0053*
Tiredness	Cost due to presenteeism	0.7149	<0.0001*
Pain		0.7202	<0.0001*
Tenderness to touch or pressure		0.8673	<0.0001*
Morning joint stiffness		0.6685	<0.0001*
Morning joint stiffness duration		0.6114	0.0002*
Tiredness	Total indirect cost	0.7997	<0.0001*
Pain		0.7428	<0.0001*
Tenderness to touch or pressure		0.8304	<0.0001*
Morning joint stiffness		0.6857	<0.0001*
Morning joint stiffness duration		0.6602	<0.0001*
Tiredness	Utility	−0.7724	<0.0001*
Pain		−0.8030	<0.0001*
Tenderness to touch or pressure		−0.7558	<0.0001*
Morning joint stiffness		−0.8488	<0.0001*
Morning joint stiffness duration		−0.7269	<0.0001*

## Discussion

Although PsA is believed to impose a high clinical and societal burden and reduce the quality of life, especially in active disease state, there is no evidence to confirm this in Poland. Moreover, PsA is known to generate significant indirect costs, mainly due to presenteeism. Our study showed a significant moderate-to-strong correlation between disease activity and indirect costs (total costs as well as those of absenteeism and presenteeism).

To our knowledge, our study is the first to measure indirect costs of PsA in Polish patients. Therefore, we deemed it necessary to assess a societal burden of PsA in Poland using a comprehensive approach. Moreover, in the absence of reliable data on the health-related quality of life and utility of patients with PsA in Poland, we believed that a study that would provide such information was urgently needed. Our project involved the assessment of the indirect costs of absenteeism and presenteeism, based on the WPAI questionnaire. The methodology used in our study allowed us to obtain novel data that fill the information gap.

Our study revealed that there may be substantial indirect costs related to PsA. The society can suffer from productivity loss reaching €484.56 per working patient per month, which may give rise to a considerable amount when taking into account the prevalence of the disease. In addition, our study showed that both disease activity and quality of life can be important factors influencing total indirect costs.

In order to compare our results with findings of other investigators, we performed a review of medical databases to identify relevant sources of data on indirect costs of PsA reported elsewhere [11]. The search was conducted using MEDLINE (via PubMed), Embase and Centre for Reviews and Dissemination databases and included original studies, systematic reviews, economic evaluations, as well as conference abstracts and posters. We identified 8 records (7 studies) presenting indirect costs of PsA from the following European countries: Spain [12], Hungary [13, 14], Germany [15], Italy [16], Czech Republic [17], Norway [18] and Hong Kong [19]. Among the identified records, there were 5 economic evaluations, 2 conference abstracts and 1 poster.

We observed a large variety of indirect resources and calculation methods used in the identified records. All of the identified studies took into account absenteeism, although its components differed. In addition, all of the identified studies included the cost of sick leave as part of indirect costs [12–19]. Early retirement (full-time disability pension) was considered in 5 studies [12–15, 17, 18]. The cost of patient assistance was considered in 2 studies [12, 16]. In addition, 1 study took into account out-of-pocket expenses incurred by patients due to PsA [12]; 1 study took into account part-time disability pension due to PsA [17]; 1 study considered the cost of days off household work due to the disease [19]; and 2 studies took into account unemployment caused by PsA [12, 18]. None of the studies considered early death or presenteeism, as PsA is not a relevant cause of premature death. Of all the identified studies, 3 used the HCA [13, 14, 16, 19], 1 used the FCA [19], and 2 provided calculations based on both approaches [15, 18]. Only 1 study did not specify the methodology for the estimation of indirect costs [12]. The number of patients varied among the identified studies from 61 [17] to 908 [15]. The euro (EUR; €) was the most common currency and was used in 6 of the studies [12–18]. Only 1 study used the US dollar (USD; $) as the currency [19]. However, all of those studies focused only on the assessment of indirect costs without assessing correlations with utility or disease activity.

In a conference abstract, Moreno et al. [12] considered indirect costs related to sick leave, early retirement, patient assistance and out-of-pocket expenses paid by the patient due to PsA. Cost data were obtained from 287 patients in Spain and expressed in euros (2008). The methodology used for the calculation of indirect costs was not specified. The mean annual indirect cost per patient was reported to reach €1261 (95 % CI €581–€1987) [1].

Brodszky et al. [13, 14] analysed indirect costs associated with sick leave and early retirement, calculated with the HCA. Cost data were obtained from 183 patents in Hungary and presented as 2007 euros; the mean annual indirect cost per patient was €2904 (SD €4899) [13, 14].

Huscher et al. [15] considered sick leave and early retirement as components of indirect costs calculated using both the HCA and FCA. Cost data were obtained from 908 patients with PsA in Germany and expressed in 2002 euros. They reported the mean annual indirect costs per patient of €1374 (SD €4447) for sick leave (calculation methodology was not specified). The average annual cost of permanent work disability was €6545 (SD €13,596) and €1040 (SD €2160) using the HCA and FCA, respectively [15].

Olivieri et al. [16] analysed sick leave and patient assistance as components of indirect costs calculated using the HCA. Cost data were obtained from 107 patients with PsA in Italy and presented as 2007 euros. The study presented a cost-effectiveness analysis of tumour necrosis factor inhibitors in the therapy of patients with PsA. The cost was presented as a baseline value of €576.30 (SD €1565.11; 95 % CI €276.33–€876.28) for 6 months [16].

In a conference poster prepared for ISPOR 2010, Klimes and Dolezal [17] reported findings on indirect costs related to sick leave, early retirement and part-time disability pensions of patients with PsA. Costs were obtained from 61 patients in the Czech Republic, calculated using the FCA and expressed in 2009 euro [17].

Kvamme et al. [18] considered the indirect costs related to sick leave, early retirement and unemployment, calculated using both the HCA and FCA. Cost data were obtained from 374 patients in Norway, including 311 patients treated with synthetic disease-modifying antirheumatic drugs and 63 patients treated with biologic disease-modifying antirheumatic drugs. Cost data were presented in 2011 euro [18]. The study compared the indirect costs for patients with PsA treated with synthetic or biologic disease-modifying antirheumatic drugs. The average productivity loss over 2 years per patient was €58,215 and €74,009 for synthetic and biologic drugs, respectively, calculated using the HCA, and the average productivity loss over 2 years per patient was €14,209 and €17,948 for synthetic and biologic drugs, respectively, calculated using the FCA.

Our study has several limitations. First, it was designed to survey a heterogeneous group of patients with PsA. The methods for the inclusion of respondents with randomised allocation might have made the results of the study fully representative for the Polish population of patients with PsA; however, a relatively small number of patients limited the results. A larger study based on the same protocol is therefore needed to provide more reliable data. Second, as there is no specific scale to assess the disease activity of PsA, we assessed the disease activity with our own questionnaire developed on the basis of an adjusted BASDAI. This may constitute another limitation because the questionnaire has not been validated.

Despite a relatively small population of patients, our findings are comparable to those from other countries. The results of our study reflect the real impact of PsA on the economic development of Poland and allow a reliable economic analysis that is required for the development of clinical practice guidelines, optimal in terms of resource allocation. The results of our study have a wide application as they can be used to inform economic models created to assess cost-effectiveness of a new treatment in Poland, increasing the odds for a positive reimbursement decision.

## Conclusions

PsA imposes a personal burden and affects the patient’s ability to work. Moreover, it generates considerable indirect costs, mainly due to lower productivity at work, as well as reduces the patients’ quality of life. Further studies, with a fully representative population of patients, are needed to elucidate the relation between the quality of life, disease activity and indirect costs of disease in patients with PsA in Poland.
